# Is immunization with palivizumab really effective in high-risk children?

**DOI:** 10.34763/jmotherandchild.20222601.d-22-00049

**Published:** 2023-02-22

**Authors:** Marjana Jerković Raguž, Tomica Božić, Tamara Nikše

**Affiliations:** Department of Neonatology. Clinic for Children's Diseases, University Clinical Hospital Mostar. Bosnia and Herzegovina Serbia; Department of Cardiology. Clinic for Children's Diseases, University Clinical Hospital Mostar. Bosnia and Herzegovina Serbia; Department of Pulmology. Clinic for Children's Diseases, University Clinical Hospital Mostar. Bosnia and Herzegovina Serbia

**Keywords:** infant, immunization, respiratory infection

## Abstract

The aim of this research is to determine the specific characteristics of the immunized children during a 15-year period and the readmissions to hospital due to potential infections of the respiratory tract.

**Material and methods:**

This retrospective cohort study was conducted in the period from October 2008 to March 2022. The test group consists of 222 infants who met the strict criteria for immunization.

**Results:**

The study observed 222 infants who were immunized with palivizumab during the 14-year period. 124 (55.9%) infants were preterm (< 32 weeks) and 69 (31.1%) were infants with congenital heart defects, whereas 29 (13.1%) exhibited other individual risk factors. 38 (17.1%) were re-admitted to the pulmonary ward. Upon re-admission, a quick test to diagnose for RSV infections was conducted and only one infant tested positive.

**Results:**

The conclusion of our 14-year study is that palivizumab prophylaxis has truly proven itself effective for infants at risk in our region during the research time period. Over the years, the immunization season has not changed and the number of doses hasremained the same, as have the indications for immunization. What has changed, however, is an increase in the number of immunized infants without a significant increase in the number of re-admissions to hospital on account of respiratory disorders.

## Introduction

Respiratory Syncytial Virus (RSV) is the primary cause of serious infections of the lower respiratory tract (LRI) in infants, and almost all infants are infected at least once by the age of 24 months [1]. Despite numerous studies, the role of the immune response in the pathogenesis of the infection has not been clarified, nor has the connection between the infection and increased reactivity of the respiratory tract [2]. Presently, the available treatments for serious LRI caused by RSV are symptomatic therapies, such as supplemental oxygen and mechanical ventilation, which makes treating serious RSV infections a challenge for healthcare providers [3]. Preterm birth, chronic pulmonary disease, congenital heart disease (CHD), an immuno compromised state, and Down Syndrome are significant risk factors for a serious RSV infection [4]. In the absence of a vaccine, the only effective method for the prevention of serious RSV infections in high-risk infants is prophylactic immunization with palivizumab, which is an anti-RSV monoclonal antibody. Therefore, the prevention of RSV infections in high-risk infants is crucial [5]. In many countries, palivizumab is recommended for the prevention of serious LRI caused by RSV in high-risk infants [6, 7]. However, although palivizumab is an effective prophylaxis, it is restricted to risk groups because of its high price. Guidelines recommend the first dose of palivizumab be given at the beginning of the RSV season. However, it is difficult to determine the beginning of the RSV season as it varies from year to year depending on geographic location [8].The administration of palivizumab in Bosnia and Herzegovina (BH), and in Mostar as well, began in 2008 through the initiative of the Neonatal Association BH. The costs of passive immunization with palivizumab of children in our hospital were covered by the Health Insurance Institute and the Fund of the Federation of BH. To ensure optimal use and relevant health institution expenditure, clinical guidance has been issued on a national basis; it differs with respect to the recommendations for the administration of immunization. On the basis of the analysis and conclusions of a national study conducted in Japan, health insurance approved additional indications for the administration of palivizumab to infants [1]. Furthermore, Spain revised its guidelines in 2013 and then again in 2019, expanding on the indications for the immunization of infants at risk [9].Thus, the indications for administering palivizumab are broad (immuno-compromised infants and infants with congenital anomalies of the respiratory tract or cystic fibrosis and neuromuscular disease) with a tendency to include other risk groups [1, 9].As there is still no effective vaccine, the American Academy of Pediatrics has issued contemporary guidelines for palivizumab prophylaxis against RSV infections in order to protect as many infants at risk as possible. The aim of prophylaxis is to reduce morbidity, without the illusion that its application will affect mortality in a statistically significant manner [10]. Due to the high costs of prophylaxis, its rational application is imperative, whereby respect for the decisions of pediatrists regarding the infants at risk, for whom the prophylaxis has been recommended will play a very big role. Thus, the aim of this research is to determine the specific characteristics of the immunized children during a 15-year period and the admissions to hospital due to potential infections of the respiratory tract.

## Material and methods

This retrospective cohort study was conducted at the Neonatal Dispensary of the Neonatal Intensive Care Unit (NICU) at the Clinic for Children’s Diseases at the UCH Mostar in the period from October 2008 to October 2022. National guidance was based on earlier research and recommendations by the AAP. All individual risk factors were given a numerical score. If the initial total score was 4 or higher, indications for prophylaxis were determined **(Table 1)**.

**Table 1:** National guidance based on earlier research and recommendations by the AAP:

Primary recommendations for immunization	Score	*Individual risk factors	Score
Preterm infants with a gestational age (GA) < 29 weeks in the RSV season	4	Neurological disorders	1
Infants with bronchopulmonary dysplasia (BPD) < 2 years of age if they were undergoing therapy for BPD in the RSV season	4	Birth weight less than 1500 g	1
Infants with a congenital heart defect (HS-CHD) < 2 years of age in the RSV season	4	Mechanical ventilation for a period of 48 hours prior to the commencement of the RSV season;	1
Preterm infants with a GA >29<32 weeks (< 6 months of the RSV season with *individual risk factors	2	Discharged in the period from November 1 to March 31	1
Immunodeficiency	4	Low social status	0.5
		Visits to kindergarten	0.5
		Twins	0.5
		Exposure to smoke	0.5

The recorded parameters for the infants were: gender, gestational age, birth weight, type and duration of therapy during the first period of hospitalization in the NICU, the risk factors which were scored for immunization, type of birth and parity of the mother, and the re-hospitalization of immunized infants. Every infant who was re-admitted for further treatment was hospitalized in the pulmonary ward of the Clinic for Children’s Diseases UCH Mostar or in one of the hospitals in the Herzegovina region (Brankovac, Livno, Konjic), for which medical records are available. The final decision on the administration of palivizumab was made by the NICU manager at UCH Mostar. Palivizumab was administered at the Neonatal Dispensary of the NICU at the Clinic for Children’s Diseases at the UCH Mostar. The NICU treats all the ill neonates in the Herzegovina region. Infants who require surgical treatment for heart defects are referred to tertiary level clinical centers (UCH Sarajevo and Vienna). A nurse contacted and informed the parent/guardian to arrange immunization. Patients who were immunized and belonged to other hospital institutions in their home cities were contactedby phone for information about possible re-hospitalization due to respiratory pathology. All the infants who had been admitted for hospitalization were quickly tested for RSV, the results of which were available on the same day at the Institute for Microbiology at the UCH Mostar. Upon admission, a quick test to diagnose for RSV infections was conducted, and only one infant tested positive. The Directigen RSV test is a quick chromatographic immunoassay for the direct and qualitative detection of RSV antigens in nasopharyngeal washes/swabs from patients who are suspected of having a respiratory infection. The test is intended for in vitro diagnosis and as an aid in the diagnosis of RSV infection in pediatric patients. As for the limitations of the study results, for all specimens evaluated, the overall sensitivity and specificity of the *Directigen RSV* test for RSV were 80% and 91% respectively, compared to culture.

The data was collected from medical histories, immunization protocols and discharge letters. Palivizumab is a recombinant, humanized monoclonal antibody produced by DNA technology in host murine myeloma cells. The representative for palivizumab (**SYNAGIS**®) for our market is Abbvie from Sarajevo, BH. A list was compiled of the number of infants who met the criteria before the commencement of the immunization season every year, and the list of previously immunized infants was revised (infants with congenital heart disease [CHD]). The parents were informed about passive immunization and signed informed consent forms for palivizumab administration. This study was approved by the Ethics Committee of Clinical Hospital Centre Mostar and valid documentation exists.

### Statistical Methods

The data was analysed via descriptive statistical methods, with categorical variables shown as frequencies and percentages, whilst continuous variables are shown as medians.

## Results

The study encompassed 222infants who were immunized with palivizumab during the14-year period. The study group also included all the immunized infants who had been re-admitted to hospital due to infections of the lower respiratory system.

Of the total of 124 preterm infants, 68 (30.6%) were born at a gestational age between 30-32^+6/7^ weeks. 145 (65.3%) immunized infants required mechanical support.

### The demographic characteristics of the infants who received passive immunization during the study period are shown in Table 2.

**Table 2:** Demographic characteristics of children who received passive immunization during the study period 2008–2022 in Clinic of Child Disease UHC Mostar.

	Children with CHD n=69		Preterm infants n=124		Children with other diagnosis n=29		Total n=222	
Male	39	56.5%	69	55.6%	20	69.0%	128	57.7%
Female	30	43.5%	55	44.4%	9	31.0%	94	42.3%
**Gestational age (weeks+days)**								
< 29 +6d	0	0.0%	47	37.9%	1	3.4%	48	21.6%
30-32 +6d	2	2.9%	64	51.6%	2	6.9%	68	30.6%
33-37 +6d	9	13.0%	13	10.5%	12	41.4%	34	15.3%
38-41 +6d	58	84.1%	0	0.0%	14	48.3%	72	32.4%
**Birthweight (g)**								
<1000	0	0.0%	21	16.9%	0	0.0%	21	9.5%
1001-1500	1	1.4%	65	52.4%	0	0.0%	66	29.7%
1501-2500	11	15.9%	37	29.8%	10	34.5%	58	26.1%
>2500	57	82.6%	1	0.8%	19	65.5%	77	34.7%
**Mechanicalventilation**								
Yes	26	37.7%	97	78.2%	22	75.9%	145	65.3%
No	43	62.3%	27	21.8%	7	24.1%	77	34.7%
**Parityofmother**								
Primiparous	31	44.9%	75	60.5%	14	48.3%	120	54.1%
Multiparous	38	55.1%	49	39.5%	15	51.7%	102	45.9%
**Pregnancy**		0.0%						
Singletones	65	94.2%	81	65.3%	26	89.7%	172	77.5%
Multiplepregnancy	4	5.8%	43	34.7%	3	10.3%	50	22.5%

Of this total, 124 (55.9%) infants were preterm (< 32 weeks) and 69 (31.1%) were infants with CHD, whereas 29 (13.1%) exhibited other individual risk factors: preterm neonates GA 33–34 weeks, BPD, neurological disorders, respiratory anomalies, and multi-organ anomalies.

### The distribution of the total number of immunized infants during the period of study is presented in Figure 1.

**Figure 1:**
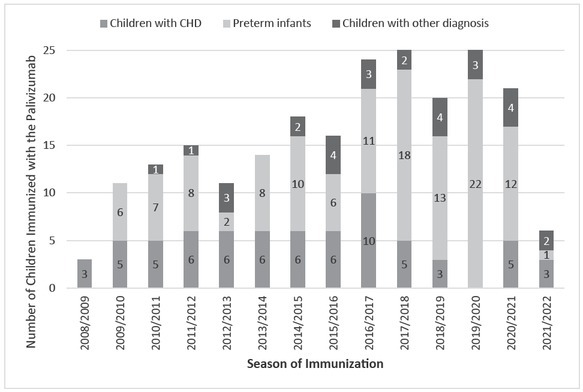
The distribution of the total number of immunized children during the period of study 2008–2022 in Clinic of Child Disease UHC Mostar.

Of the 222 infants immunized, 38 (17.1%) were re-admitted to the pulmonary ward for respiratory system infections. Of the total re-hospitalized infants, 21 (55.3%) had pneumonia, 16 (42.1%) had bronchiolitis, and only one had laryngitis. They were treated with antibiotic therapy in the cases of pneumonia, and with symptomatic therapy in the case of bronchiolitis.

A male infant was treated for pneumonia caused by RSV in the pulmonology department of our hospital in 2017. The boy was a chronic patient being treated for *Osteogenesis imperfecta* and had received all five doses of Palivizumab prior to infection. For most of the infants, re-treatment lasted between 3–5 days, while nine of the infants remained in the hospital longer than 5 days. 50% of the re-admitted infants were under the age of six months and were treated for less than a week on average. All immunized children who were re-treated for pulmonary pathology during the COVID-19 pandemic were tested for COVID-19 and the result was negative.

**Figure 2:**
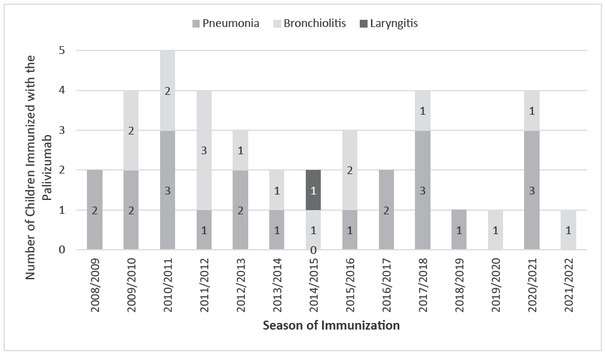
The number of immunized children readmitted for infections of the lower respiratory system during the study period 2008–2022 in Clinic of Child Disease UHC Mostar.

***The number of immunized infants re-admitted for infections of the lower respiratory system during the study period 2008-2022 is presented in Figure 2***.

## Discussion

In industrialized and developing countries, RSV infections are the leading cause of death associated with respiratory infections [11]. Although many studies on the pathogenesis of RSV infections have brought new discoveries, we are still unable to form a clear and complete picture. This results in limited therapeutic options, which include primary support measures, inhalation with unconfirmed effectiveness, and medicine with a poor safety and dubious efficacy profile [2]. RSV infections in risk groups of infants are frequently accompanied by the development of various acute complications, and later in life, the infection often leads to labored breathing and asthma [12]. Such clinical presentations of infections most often require hospitalization, very often in intensive care units; sometimes, they even require mechanical ventilation [13]. Palivizumab represents an effective prophylaxis, but because of its price, it is limited to the risk groups, particularly in countries such as ours, where strict criteria and guidelines exist for the immunization of infants at risk. However, a study published in Japan in 2021 concludes that the indications for palivizumab should be expanded to pediatric patients with illnesses connected to reduced ventilatory capacity or difficulties with productive coughing [14]. The efforts that experts are putting into the production of a vaccine against the RSV for children and pregnant women gives hope that the morbidity and mortality caused by RSV infections may be significantly decreased in the near future [15]. During the 14-year period, 222 infants were immunized at the Clinic for Children’s Diseases UCH Mostar, all of whom fulfilled individual criteria which have been accepted by the Neonatal Association of BH. This constitutes 0.88% of the total live births (approximately 25,000) in the period of the study. Over the 14years, there was an increase in the number of infants who received palivizumab, which corresponds to the results of other studies [16], but the number of patients readmitted to the hospital remained the same throughout the period of immunization. Our study shows that 38 (17%) of the infants were treated again for respiratory illnesses, but just one child tested positive for RSV, whilst the Japanese study showed that of the 498 immunized infants, 10 returned with RSV infections [17]. This indicates that palivizumab significantly reduced the incidence of severe RSV infections, and simultaneously reduced the costs of treatment. The key success of palivizumab lies in the time of initial immunization and the number of doses, which we administer five times from October to April during the RSV infection season, whilst others begin immunization during July when the RSV season starts [18].The failure of prophylactic palivizumab to prevent illness is linked to inappropriate dosage intervals, exposure to high concentrations of the virus, the infant’s weak state, or a co-infection with other respiratory pathogens [19]. A study conducted in Spain confirms the effectiveness of the prophylaxis at 70% [20], whilst our study indicates an effectiveness of over 80%in immunized children. The decrease in the number of hospitalizations does not only lead to a reduction in costs and health institution expenditure but it also reduces the emotional and psychological results of hospitalization, which is not only a burden for the child, but also for the parents.

## Conclusion

The conclusion of our 14-year study is that palivizumab prophylaxis has truly proven itself effective for infants at risk in our region during the research time period. Over the years, the immunization season has not changed, and the number of doses has remained the same, as have the indications for immunization. What has changed, however, is an increase in the number of immunized infants without a significant increase in the number of re-admissions to hospital on account of respiratory disorders. This is evidence that the recommendations and guidelines for palivizumab prophylaxis are effective for the time being in our environment, and that immunization is the only preventive measure and safe way to fight RSV. Also, as re-hospitalized immunized children did not have RSV infection, this significantly facilitated the course, method and length of treatment. This reduces the psychological trauma for the family, and contributes to the reduction of hospital treatment costs, which is a very important factor in favor of immunization in a poor country such as ours.
